# Regulation of chloroplast and nucleomorph replication by the cell cycle in the cryptophyte *Guillardia theta*

**DOI:** 10.1038/s41598-017-02668-2

**Published:** 2017-05-24

**Authors:** Ryo Onuma, Neha Mishra, Shin-ya Miyagishima

**Affiliations:** 1 Department of Cell Genetics, National Institute of Genetics, Yata 1111, Mishima, Shizuoka 411-8540 Japan; 2 Department of Genetics, Graduate University for Advanced Studies (SOKENDAI), Mishima, Shizuoka 411-8540 Japan

## Abstract

The chloroplasts of cryptophytes arose through a secondary endosymbiotic event in which a red algal endosymbiont was integrated into a previously nonphotosynthetic eukaryote. The cryptophytes retain a remnant of the endosymbiont nucleus (nucleomorph) that is replicated once in the cell cycle along with the chloroplast. To understand how the chloroplast, nucleomorph and host cell divide in a coordinated manner, we examined the expression of genes/proteins that are related to nucleomorph replication and chloroplast division as well as the timing of nuclear and nucleomorph DNA synthesis in the cryptophyte *Guillardia theta*. Nucleus-encoded nucleomorph *HISTONE H2A* mRNA specifically accumulated during the nuclear S phase. In contrast, nucleomorph-encoded genes/proteins that are related to nucleomorph replication and chloroplast division (FtsZ) are constantly expressed throughout the cell cycle. The results of this study and previous studies on chlorarachniophytes suggest that there was a common evolutionary pattern in which an endosymbiont lost its replication cycle-dependent transcription while cell-cycle-dependent transcriptional regulation of host nuclear genes came to restrict the timing of nucleomorph replication and chloroplast division.

## Introduction

Chloroplasts trace their origin to a primary endosymbiotic event in which an ancestral cyanobacterial endosymbiont was reduced into the chloroplast (the primary chloroplast surrounded by the inner and the outer envelope membranes). The ancient alga which resulted from this primary endosymbiotic event evolved into the Glaucophyta (glaucophyte algae), Rhodophyta (red algae) and Viridiplantae (the chlorophyte algae, streptophyte algae and land plants). Chloroplasts then spread into other lineages of eukaryotes through secondary endosymbiotic events in which a red or a green alga became integrated as secondary chloroplasts into a previously nonphotosynthetic eukaryote. The secondary endosymbiotic event of a red alga gave rise to chloroplasts in stramenopiles (diatoms, brown algae, etc.), haptophytes, cryptophytes, most of the photosynthetic dinoflagellates, and apicomplexans. The euglenids and chlorarachniophytes possess chloroplasts of a green algal secondary endosymbiotic origin. The question of exactly how many endosymbiotic events have given rise to this evident diversity remains unanswered^1, 2^.

**Figure 1:**
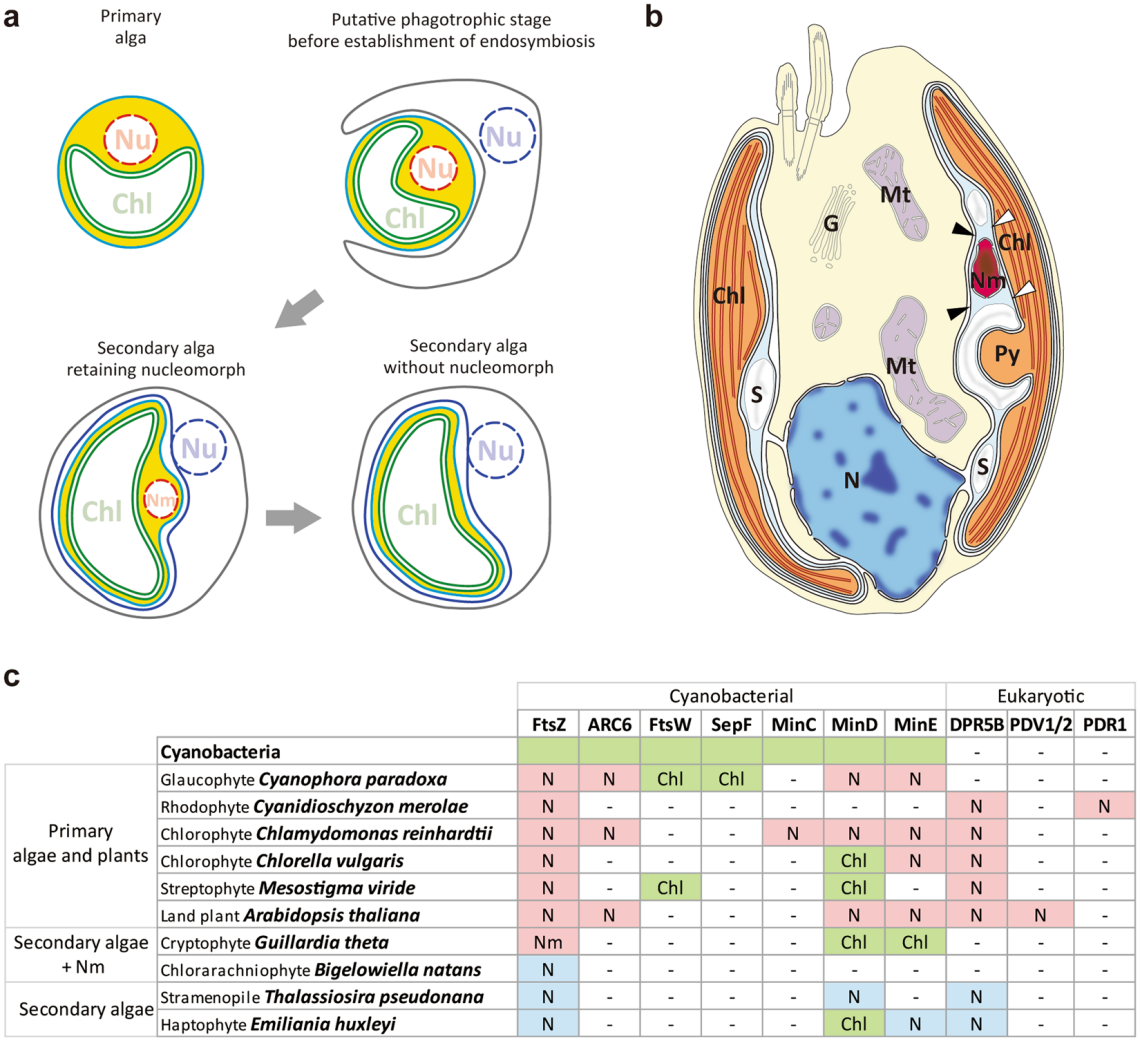
A diagram showing the course of the establishment of secondary chloroplasts, illustration of the cryptophyte *Guillardia theta* and distribution of chloroplast division proteins in eukaryotes. (**a**) Schematic view of organelle reduction and membrane heredity through secondary endosymbiosis. The green double membrane, sky-blue membrane and blue membrane represent the inner and the outer chloroplast envelope membranes, periplastidal membrane and chloroplast ER, respectively. The space indicated in yellow is the periplastidal compartment (PPC), which is homogeneous with the cytoplasm in primary endosymbiotic algae. The evolutionary stage of cryptophytes and chlorarachniophytes corresponds to secondary alga that possesses a nucleomorph. (**b**) Schematic drawing of the ultrastructure of *G*. *theta* based on McKerracher & Gibbs (1982). Chl, chloroplast; N, nucleus; Nm, nucleomorph; Py, pyrenoid; S, starch; Mt, mitochondria; G, Golgi body. The nucleomorph is located in the PPC between the outer chloroplast envelope (white arrowheads) and the periplastidal membrane (black arrowheads). (**c**) Distribution of chloroplast division proteins that are descended from the cyanobacterial ancestor of chloroplasts and the eukaryotic host (based on Miyagishima *et al*.^10^ and Hirakawa & Ishida 2015). The “Chl”, “N” and “Nm” in each column indicate that the gene is encoded in the chloroplast, nucleus and nucleomorph genome, respectively. The hyphen indicates that the gene is not found in the corresponding organism. Proteins encoded in the cyanobacterial or chloroplast genome are boxed in green, proteins encoded in the nuclear genome of primary algae or plants are boxed in pink and proteins encoded in the nuclear genome of secondary algae are boxed in blue. The accession numbers of the amino acid sequences are listed in Supplementary Table 1.

During the course of the establishment of these secondary chloroplasts, most of the eukaryotic algal endosymbiont cellular compartments other than the chloroplast and plasma membrane had been lost. This reduction in the number of the cellular compartments of the endosymbionts was believed to have resulted in the establishment of the present secondary chloroplasts which are typically surrounded by four (or three) membranes. The inner two membranes are descended from the inner and the outer envelopes of the primary chloroplast. The two additional membranes are thought to correspond to the plasma membrane of the endosymbiotic eukaryotic alga and the phagosomal membrane of the host cell, respectively (Fig. 1a). Between the inner two membranes and the second outermost membrane (called the “periplastidal” membrane), there is a small space called the “periplastidal compartment” (PPC) which is comprised of the reduced cytoplasm of the endosymbiotic eukaryote. The degree of reduction in the eukaryotic algal endosymbiont differs depending on the lineage, especially in terms of the presence of the nucleomorph in the PPC, which is a relic nucleus of the eukaryotic endosymbiont. Cryptophytes and chlorarachniophytes possess a nucleomorph of a red algal or a green algal endosymbiotic origin, respectively (Fig. 1b). In contrast, other secondary algae have completely lost the nuclei of endosymbionts^2, 3^.

The continuity of both primary and secondary chloroplasts is maintained by chloroplast division in eukaryotic host cells. The majority of algal species have one or at most only a few chloroplasts per cell. Thus chloroplast division takes place once per host cell cycle. In addition, as in the case of chloroplasts, the nucleomorph is also replicated once per host cell cycle and inherited by a daughter chloroplast and cell^4–7^. This synchronization of endosymbiotic cell/chloroplast division with the host cell cycle is believed to have enabled the host cells to permanently inherit the primary or secondary endosymbionts/chloroplasts.

In primary algae and land plants, chloroplast division is performed by the constriction of a macromolecular ring-like division machinery that is comprised of a self-assembling GTPase FtsZ of cyanobacterial endosymbiotic origin and another, self-assembling GTPase dynamin (DRP5B) of eukaryotic host origin^8, 9^. Prior to chloroplast division, the FtsZ ring forms on the stromal side of the provisional chloroplast division site with the assistance of certain FtsZ regulatory proteins such as MinD, MinE and ARC6, followed by the formation of the inner PD ring of unknown molecular composition on the stromal side. Then the glucan-based outer PD ring, which is synthesised by the PDR1 protein, forms on the cytosolic side. Finally, DRP5B is recruited to the cytosolic side of the division site and the competent chloroplast division machinery begins to constrict^8, 9^.

We previously showed in primary algae (the glaucophyte, red, chlorophyte and streptophyte algae), that chloroplast division initiates in the S phase and that some of the nucleus-encoded components of the chloroplast division machinery are specifically expressed during the S phase^10^. In contrast to the nucleus-encoded division genes, it was shown that the chloroplast-encoded division genes are constantly expressed throughout the host cell cycle^10^. These results suggest that the onset of chloroplast division is restricted to the S phase by the host cell cycle at the host nuclear transcriptional level and that the chloroplast has lost such division-cycle-dependent transcriptional regulation.

In the case of the secondary chloroplasts, FtsZ is encoded in the nuclear genome of stramenopiles, haptophytes and chlorarachniophytes (Fig. 1c). It was shown in the chlorarachniophyte *Bigelowiella natans* that the nucleus-encoded FtsZ localizes at the chloroplast division site^11^. In cryptophytes, FtsZ is not encoded in the nuclear genome, but rather, in the nucleomorph genome^12^. In stramenopiles, nucleus-encoded DRP5B of red algal endosymbiotic origin localizes at the chloroplast division site^8^. In addition, the outer PD ring has been observed on the outer side of the second innermost membrane^13, 14^. Thus, the division of the inner pair of membranes in secondary chloroplasts involves at least a portion of the primary chloroplast division machinery that is descended from the endosymbiotic alga.

In the stramenopile *Seminavis robusta*, the nucleus-encoded *FtsZ* mRNA accumulates in a specific period of the 24-h light/dark cycle in which the chloroplasts divide. Thus, it is most likely that chloroplast division is restricted to the host S/G2 phase by the host nuclear transcriptional level^15^. However, in the case of algae that have retained a nucleomorph, the situation becomes theoretically more complicated, although algae without nucleomorph, such as stramenopiles, also should have evolved through such a stage. In this case, the eukaryotic cell cycle in the nucleomorph became linked with the host cell cycle. Chloroplast division, which was originally regulated by the nucleus of the endosymbiont, became linked with the host cell cycle.

In the chlorarachniophyte *B*. *natans*, a recent study showed that host nucleus-encoded histone proteins and DNA-polymerases are targeted into the nucleomorph. The mRNAs of respective genes accumulate specifically around the time of the host S phase when the nucleomorph is replicated, while all of the nucleomorph-encoded replication-related genes are expressed throughout the cell cycle. Thus it is suggested that nucleomorph replication is restricted to the S phase by the host nuclear transcriptional level in the chlorarachniophyte^16, 17^. The *B*. *natans* nucleomorph genome does not encode any known chloroplast division proteins, whereas two FtsZ proteins are encoded in the nuclear genome. Both of the *FTSZ* mRNA levels oscillated when the cells were synchronized by a 24-h light/dark cycle, but the peaks of the mRNA levels were not consistent with the timing of chloroplast division. In addition, both of the FtsZ proteins localize at the chloroplast division site throughout the cell/chloroplast division cycle. Thus, it is still unclear how the timing of chloroplast division is linked to the host cell cycle in chlorarachniophytes^11^. In addition, there has been no information reported on other nucleomorph-possessing algae cryptophytes in terms of regulation of chloroplast division by the host cell cycle.

In this study, we examined the expression patterns of the host cell, nucleomorph and chloroplast division-related genes/proteins in the cryptophyte *Guillardia theta* and compared the results with the results reported in other algal lineages. The results suggest that (1) the nucleomorph genome has lost its replication-cycle-regulated gene expression system, (2) the cell-cycle-regulated expression of nucleomorph-targeted proteins probably restricts the timing of nucleomorph replication as in the case of chlorarachniophytes, although nucleomorphs and chloroplasts were established independently in cryptophytes and chlorarachniophytes and (3) the nucleomorph-encoded *FTSZ* and chloroplast-encoded division genes are expressed throughout the cell cycle. Thus, there is a common evolutionary trend: endosymbiotic genomes (chloroplasts and nucleomorphs) have lost division-cycle-regulated gene expression, while the host nuclear genomes have acquired cell-cycle-linked expression of genes that are related to the division of endosymbionts.

## Results

### Selection of mRNAs and proteins for characterization of the relationship between the host cell cycle, nucleomorph replication and chloroplast division

Currently, the draft nuclear genome sequence of the cryptophyte *G*. *theta* CCMP2712 is available^18^. In addition, the nucleomorph genomes of four cryptophytes have been fully sequenced. They are *G*. *theta* CCMP2712^19^, *Hemiselmis andersenii* CCMP644^20^, *Cryptomonas paramecium* CCAP977/2A^21^ and *Chroomonas mesostigmatica* CCMP1168^22^. Each nucleomorph genome encodes approximately 500 genes in three chromosomes, including the *FTSZ* gene^12^.

The aim of this study was to characterise the relationship between the progression of the host cell cycle, timing of nucleomorph replication and timing of chloroplast division. To determine the status of these three processes in synchronized cells and to examine whether expression of the respective mRNA/proteins are regulated by the host cell cycle, we chose *G*. *theta* as a material and selected mRNAs (quantified by quantitative RT-PCR) and proteins (quantified by immunoblotting) that are related to the cell cycle, nucleomorph replication and chloroplast division, as described below.

For the host (nuclear) cell cycle, nucleus-encoded *HISTONE H2A*, *CYCLIN A* and *CYCLIN B* mRNA were chosen. Histone genes, including the *H2A* gene, are transcribed specifically during the S phase in eukaryotes^23^. *CYCLIN A* and *CYCLIN B* mRNA are accumulated specifically during the S and M phase, respectively^24^. We also chose histone H3 phosphorylated at serine 10 (H3S10ph), as an M-phase marker that is detectable with specific antibodies. For the nucleomorph replication cycle, nucleus-encoded nucleomorph-targeted *HISTONE H2A* mRNA (see below), nucleomorph-encoded *CYCLIN B* and *HISTONE H2B* mRNA were chosen. We also examined the *CDC2* (encoding a cyclin-dependent kinase) and *TUBA* (encoding alpha-tubulin) mRNA levels. Regarding chloroplast division, all of the orthologs of the known chloroplast division genes/proteins were chosen (Fig. 1c). These include the nucleomorph-encoded FtsZ protein and its mRNA as well as chloroplast-encoded *minD* and *minE* mRNA. The *G*. *theta* nuclear genome does not encode any putative orthologs of the known chloroplast division proteins.

Previous studies reported that the nucleomorph encodes a FtsZ protein of a red algal origin^19–22^. However, it has not been examined whether the FtsZ is targeted to the chloroplast division site. In this study, we prepared an anti-FtsZ antibody and showed that the FtsZ localizes at the chloroplast division site, as described below.

### Identification of the nucleus-encoded histone H2A that is targeted into the nucleomorph

**Figure 2:**
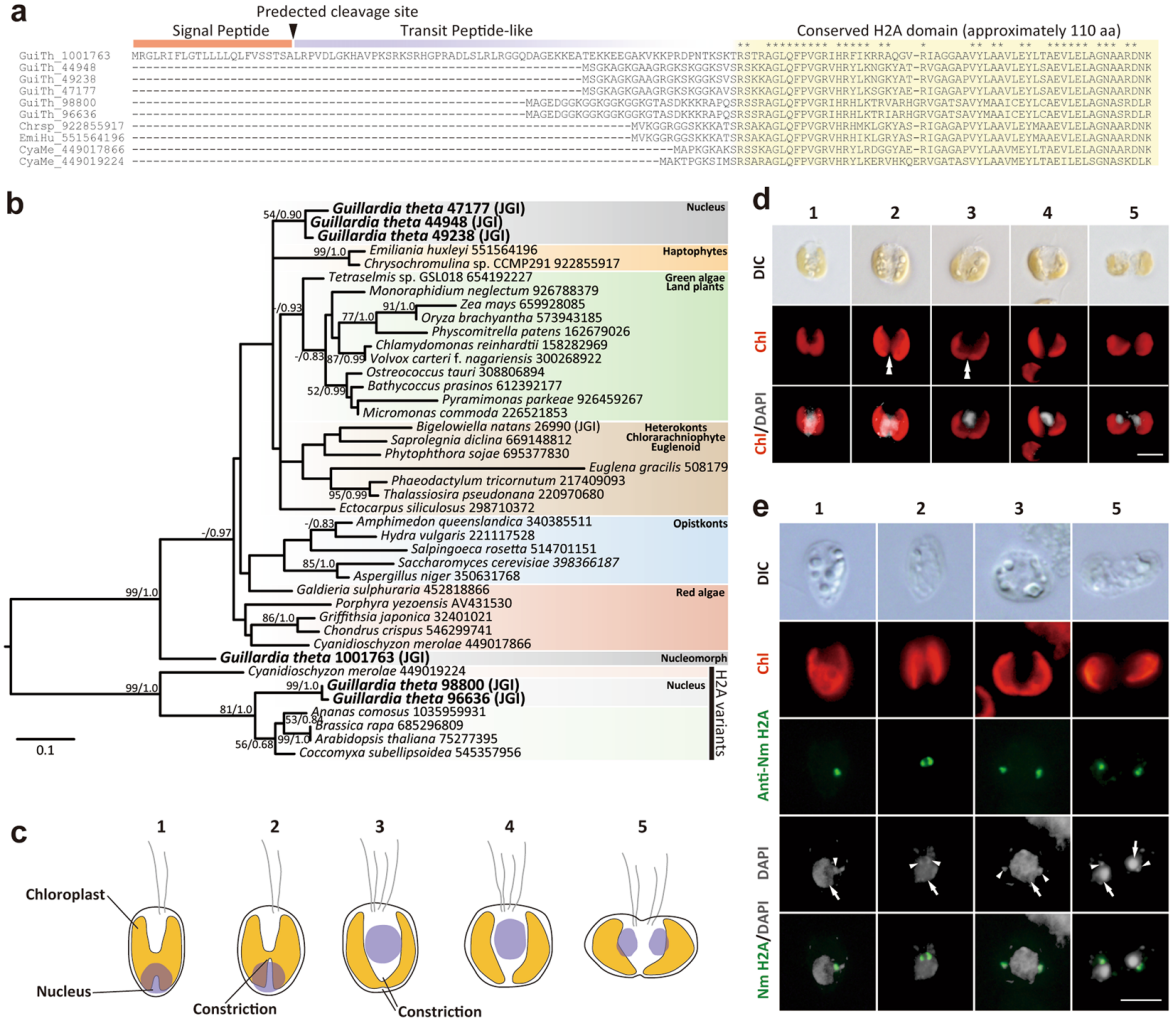
Identification of the nucleus-encoded nucleomorph-targeted histone H2A in *G*. *theta*. (**a**) Alignment of the N-terminal sequences of histone H2A proteins. The N-terminal of the *G*. *theta* histone H2A (GuiTh_1001763) is composed of a signal peptide and a transit peptide-like sequence. No signal peptide is predicted in other *G*. *theta* nucleus-encoded histone H2A (GuiTh_44948, GuiTh_49238 and GuiTh_47177) or histone H2A variants (GuiTh_98800 and GuiTh_96636). Histone H2A sequences of the haptophyte *Chrysochromulina* sp. CCMP2919 (GI#922855917), the haptophyte *Emiliania huxleyi* (GI#551564196), and the red alga *Cyanidioschyzon merolae* (GI#449017866 and GI#449019224) were also used for the alignment. The asterisk indicates an amino acid that is identical in all sequences and the conserved region is boxed in yellow. (**b**) Phylogenetic tree of histone H2A inferred from 105 amino acid sequences. The histone H2A sequences of *G*. *theta* are indicated in bold and boxed in gray. H2A variants were used as the outgroup. The bootstrap value and Bayesian posterior probability are shown at the node when the values exceed 50% (bootstrap) or 0.80 (posterior probability). The JGI accession number or GI number is shown on the right side of the species name. (**c**) Schematic drawing of the classification of the cell cycle stages based on the localization of the nucleus and the shape of the chloroplast. (**d**) DAPI-staining images showing the representative cells of the stages 1 to 5. DIC, images of differential interference contact; Chl, chloroplast autofluorescence; Chl/DAPI, merged images of Chl and DAPI. The double arrowhead indicates constriction of the chloroplast division site. Scale bar = 5 µm. (**e**) Immunofluorescent micrographs showing the localization of the nucleus-encoded nucleomorph H2A. DIC, images of differential interference contact; Chl, chloroplast autofluorescence; Anti-Nm H2A, nucleomorph H2A detected with the antibody; DAPI, DNA stained with DAPI; H2A/DAPI, merged images of H2A and DAPI. The arrow and arrowhead indicate DAPI signal of nucleus and nucleomorph, respectively. Scale bar = 5 µm.

In cryptophytes, the four sequenced nucleomorph genomes, including that of *G*. *theta*, encode histone H2B, H3 and H4, but not the H2A protein. The nuclear genome of *G*. *theta* encodes four H2A proteins, and the predicted amino acid sequence of one H2A protein (hereafter, nucleomorph H2A) contains an N-terminal extension that is predicted to have an N-terminal bipartite targeting sequence consisting of a signal peptide and a transit peptide-like sequence (Fig. 2a). The signal peptide is involved in co-translational targeting to the ER lumen. The transit peptide-like sequence lacks an aromatic amino acid such as phenylalanine at the +1 position, which is necessary for delivering proteins from the ER into chloroplast stroma in cryptophytes^25, 26^. Thus, this nucleus-encoded H2A is most likely targeted to the nucleomorph, as in the case of the nucleus-encoded nucleomorph-targeted H2A in chlorarachniophyte^16^. The three other H2A proteins (hereafter, nuclear H2A) do not contain any N-terminal extension (Fig. 2a) and formed a monophyletic group in the phylogenetic tree, although the statistical support was relatively weak (Fig. 2b; the posterior probability was 0.90). The nucleomorph H2A did not form any statistically reliable clade with any other OTUs, as in the case of the chlorarachniophyte nucleomorph H2A^16^ (in our analyses, it was omitted to avoid a long branch artifact, because the protein appears to have rapidly evolved). Thus, the evolutionary origins of the nucleus-encoded nucleomorph H2A in cryptophytes and chlorarachniophytes are unclear.

Before characterizing the localization of the nucleus-encoded nucleomorph H2A by immunofluorescence microscopy, we classified the cell cycle stages based on the localization of the nucleus and the shape of chloroplast (stages 1 to 5 in Fig. 2c,d). The progression from the stage 1 to the stage 5 is consistent with a previous observation by electron microscopy^4^. The interphase *G*. *theta* cell possesses a nucleus located posteriorly and a single cup-shaped chloroplast, which has a cleavage at the dorsal side of the cell, resulting in two lateral lobes connected by a broad bridge (The H-shape is rounded to form a cup-shape) (stage 1). In the S phase, the dorsal bridge of the chloroplast starts to constrict (stage 2). In the M phase, constriction of the dorsal bridge of the chloroplast becomes evident and the nucleus migrates to the anterior side (stage 3). After that, chloroplast division completes (stage 4). Finally nuclear division completes and cytokinesis commences (stage 5) (Fig. 2c,d).

In order to examine whether the putative nucleus-encoded nucleomorph H2A is targeted to the nucleomorph, we prepared an antibody and examined the protein localization by immunofluorescence microscopy. In interphase cells, the protein localized as a particle in the middle of the cell (Fig. 2e, stage 1). During chloroplast division, the protein localized as two particles (Fig. 2e, stages 2 and 3). During cytokinesis, one of the two particles was inherited by each daughter cell (Fig. 2e, stage 5). Throughout the cell cycle, the fluorescent signal was overlapped with the DAPI fluorescence of the nucleomorph (Fig. 2e). These results indicate that the nucleus-encoded H2A is indeed targeted to the nucleomorph.

### Regulation of the timing of nucleomorph replication by the host cell cycle at the level of nuclear transcription

**Figure 3:**
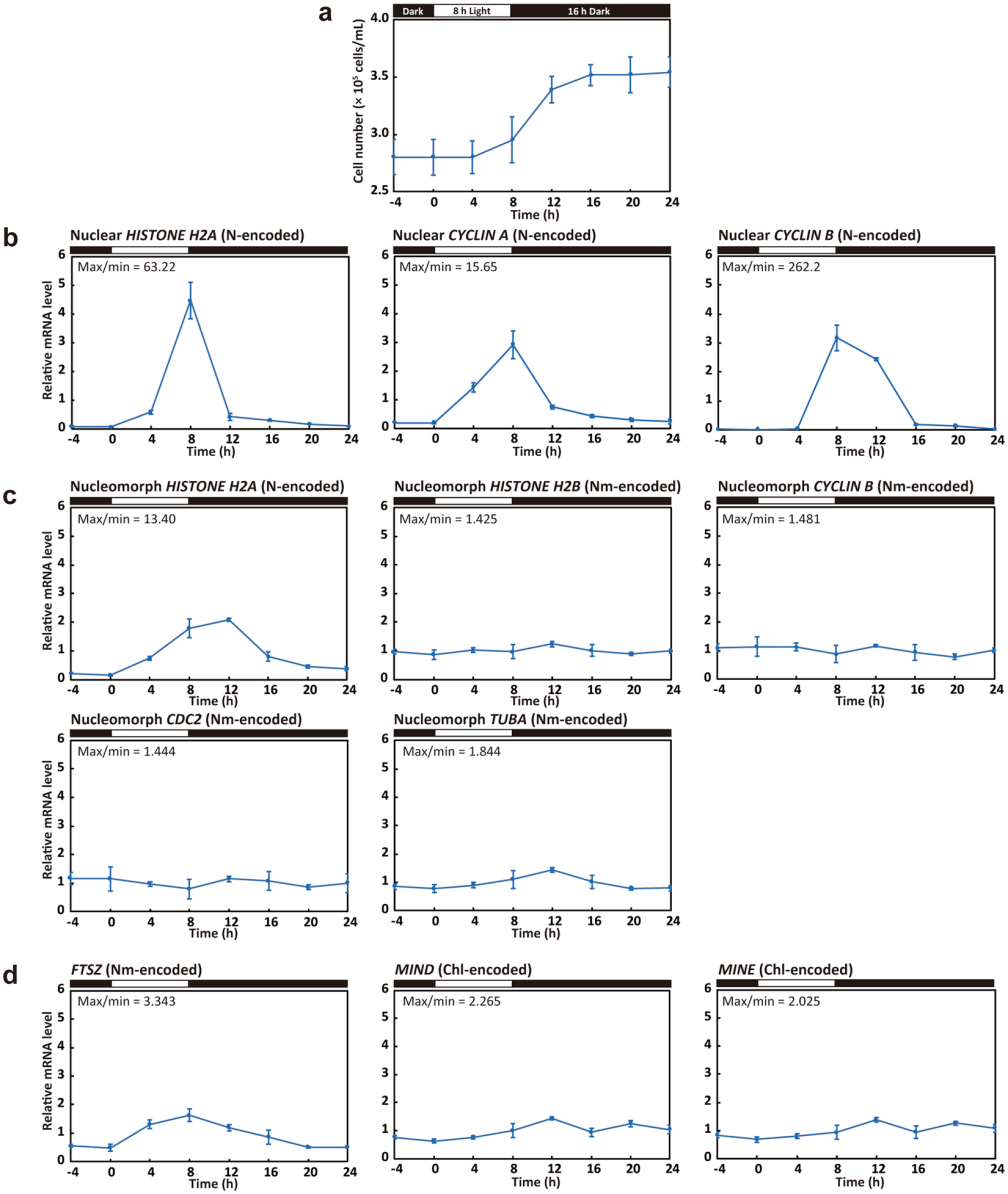
Change in the mRNA levels of the cell cycle marker genes and genes related to nucleomorph replication and chloroplast division in the synchronous *G*. *theta* culture. (**a**) Change in the cell density in the culture synchronized by the 8-h light/16-h dark cycle. The error bar represents standard deviation of six technical replicates. (**b**–**d**) Quantitative RT-PCR analyses showing the change in the mRNA levels of indicated genes. 18S ribosomal RNA was used as the internal control. The average value from hour 0 to 20 in the control culture was defined as 1.0 for each mRNA. The error bar represents standard deviation of three technical replicates. mRNA levels of the S-phase markers nuclear *HISTONE H2A* and *CYCLIN A* and M-phase marker *CYCLIN B* are shown in (**b**). mRNA levels of the nucleus-encoded nucleomorph *HISTONE H2A* and nucleomorph-encoded *HISTONE H2B*, C*YCLIN B*, *CDC2* and *TUBA* are shown in (**c**). mRNA levels of the chloroplast division genes nucleomorph-encoded *FTSZ* and chloroplast-encoded *MIND* and *MINE* are shown in (**d**). “N-encoded”, “Nm-encoded” and “Chl-encoded” indicate nucleus-, nucleomorph- and chloroplast-encoded genes, respectively. “Max/min” indicates the ratio between maximum and minimum values from hour 0 to 24. A biological replicate of this experiment is shown in Supplementary Fig. 1.

To examine the relationship between host cell cycle progression and the timing of nucleomorph replication and chloroplast division in *G*. *theta*, we developed a procedure for synchronizing the cell cycle by subjecting the culture to an 8-h light/16-h dark cycle (Fig. 3a). In this culture condition, the S-phase mRNA markers nuclear *H2A* and *CYCLIN A* peaked at hour 8 (the onset of the light period is defined as hour 0), then the M-phase mRNA marker *CYCLIN B* peaked at hour 8 and 12 (Fig. 3b). These results suggest that cells synchronously entered into the S phase and then the M phase in the culture. The cell number increased from hour 8 to 16 (Fig. 3a). The total increase in the cell number during one round of 8-h light/16-h dark was ~1.3 times. Thus ~30% of the cells underwent synchronous cell division, but the remaining 70% stayed in the G1 phase, probably because the cell growth during the light period was insufficient for these cells to enter the S phase.

Then we examined whether the replication-related genes that function in nucleomorph exhibit cell-cycle-stage specific expression (Fig. 3c). The change in the mRNA level of the nucleus-encoded nucleomorph *H2A* (peaking at hour 8 and 12; Fig. 3c) was similar to that of the S-phase markers nuclear *H2A* and *CYCLIN A* (Fig. 3b) in the synchronous culture. Thus, the expression of nucleomorph *H2A* is limited to a period around the nuclear S phase but the duration of nucleomorph *H2A* expression is longer than that of nuclear *H2A*.

In contrast to the nucleus-encoded nucleomorph *H2A*, the mRNA level of nucleomorph-encoded *H2B* was almost constant throughout the course of synchronous culture (Fig. 3c). This was also the case for nucleomorph-encoded *CYCLIN B*, *CDC2* and alpha-tubulin mRNAs (Fig. 3c), in contrast to nuclear *CYCLIN B* expression, which was restricted to M phase (hour 8 and 12). These results suggest that the timing of nucleomorph replication is regulated by the nucleus at the transcriptional level, but the nucleomorph has lost its capacity for replication-cycle-linked transcriptional regulation of replication-related genes.

**Figure 4:**
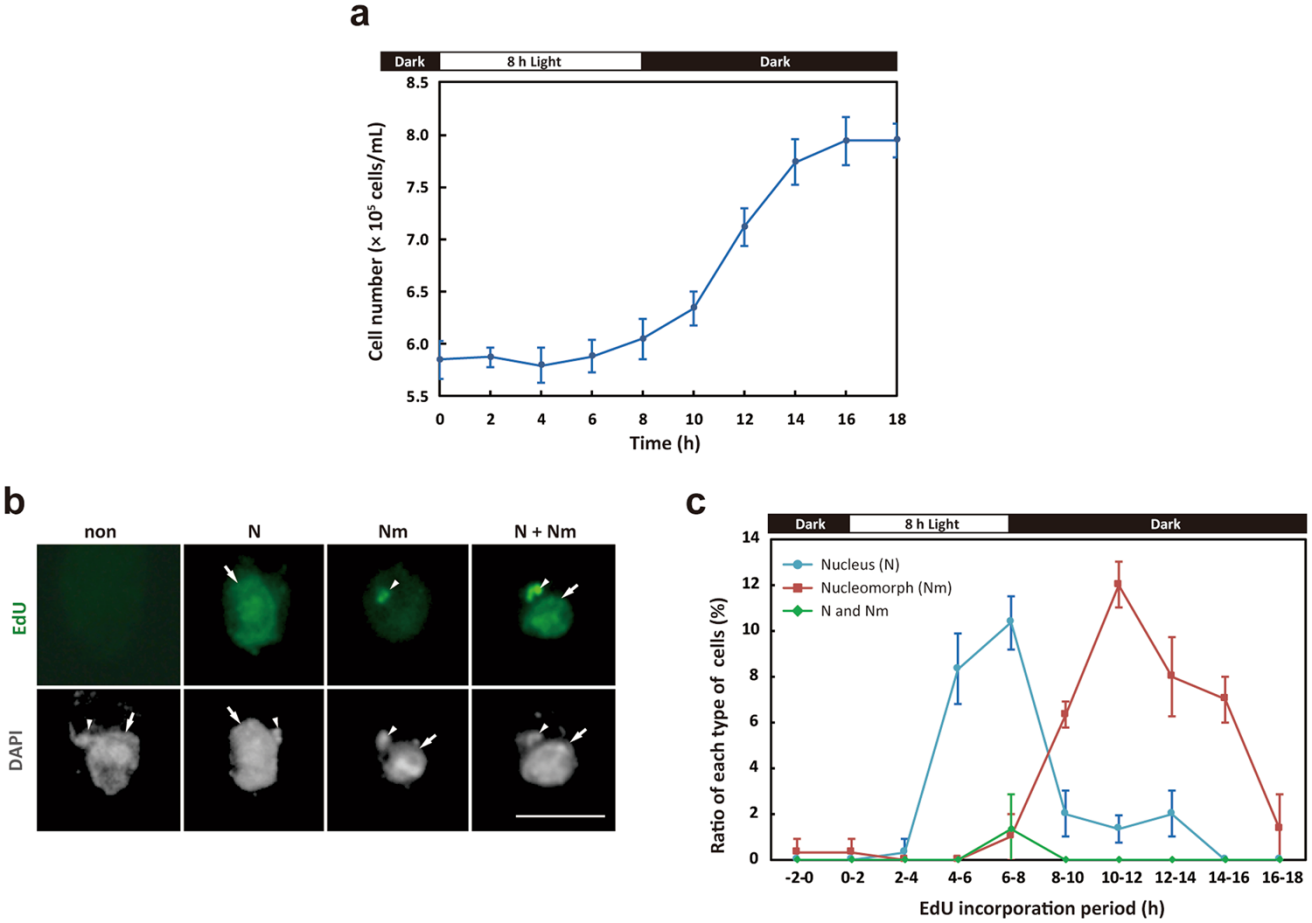
The timing of nucleus and nucleomorph DNA replication in the synchronous *G*. *theta* culture. (**a**) Change in the cell density in the culture synchronized by the 8-h light/16-h dark cycle. The error bar represents standard deviation of six technical replicates. (**b**) Fluorescent micrographs showing the EdU-labelled nucleus and nucleomorph. DIC, images of differential interference contact; DAPI, DNA stained with DAPI. The arrow and arrowhead indicate signals of nucleus and nucleomorph, respectively. Scale bar = 5 µm. (**c**) Change in the frequency of cells with EdU-labelled nucleus (blue), cells with nucleomorph (red), and cells with both EdU-labelled nucleus and nucleomorph (green). The error bar indicates standard deviation of three technical replicates.

Then to examine the timing of DNA synthesis in the nucleus and nucleomorph, we pulse labelled (two hours) the cells with 5-ethynyl-2′-deoxyuridine (EdU) every two hours in the synchronous culture. Fluorescence microscopy showed that EdU was incorporated into nuclear and/or nucleomorph DNA in some cells but not into the chloroplast DNA (Fig. 4a,b). In the assay, we found four types of cells, namely, cells with no EdU incorporation and cells in which EdU was incorporated into nuclear and/or nuleomorph DNA (Fig. 4a,b).

EdU-labelled nucleus was observed at hour 4–14 and the frequency peaked at hour 6–8 (Fig. 4c). At hour 6–8, the cells with both labelled nucleus and nucleomorph were observed at low frequency (Fig. 4c). In contrast to the nucleus, EdU-labelled nucleomorph was observed at hour 6–18 and the frequency peaked at hour 10–12 (Fig. 4c). These results suggest that nucleomorph DNA replication initiates in the late S-phase and that duration of the nucleomorph DNA replication is longer than that of the nuclear DNA replication in accord with the change in the nuclear *H2A* (Fig. 3b) and nucleus-encoded nucleomorph *H2A* mRNA levels (Fig. 3c).

### Regulation of the timing of chloroplast division by the host cell cycle at the transcriptional and translational levels

We then investigated whether chloroplast division genes/proteins exhibit cell-cycle-stage specific expression patterns. The mRNA levels of chloroplast-encoded *minD* and *minE* kept increasing during the synchronous culture but only slightly, and time-dependent accumulation was not observed (Fig. 3d). The nucleomorph-encoded *FtsZ* mRNA increased during the light period and then decreased during the dark period. However, the magnitude of the oscillation was much smaller than that of the S-phase markers nuclear *H2A* and *CYCLIN A* (Fig. 3b,d). To examine whether the oscillation of nucleomorph-encoded *FtsZ* mRNA level is linked to the host cell cycle, we examined the effect of S-phase arrest by FdU treatment (100 µg/mL; an inhibitor of DNA synthesis; added to the culture at hour 0 in G1 phase) on the change in the mRNA level. When FdU was added, the cell number did not increase (Supplementary Information, Fig. S2a). *CYCLIN A* mRNA maintained a high level of expression even after hour 12 and the magnitude of increase in *H2A* mRNA was lowered (Supplementary Information, Fig. S2b). The M-phase marker *CYCLIN B* was not detected in the FdU-treated culture (Supplementary Information, Fig. S2b). These results suggest that the cell cycle progression was arrested in the early S phase by FdU treatment. In addition, cells with two divided chloroplasts never appeared in the FdU-treated culture (Supplementary Information, Fig. S2c). In contrast, the *FTSZ* mRNA level in the S-phase-arrested culture with FdU exhibited the same change as in the control culture (Supplementary Information, Fig. S2b). This contrasts with cell-cycle-regulated genes, the expression patterns of which were altered by FdU (Supplementary Information, Fig. S2b). Thus, the change in the nucleomorph-encoded *FtsZ* mRNA was not linked to cell cycle progression, and probably was caused by the light/dark change (or cellular growth in the light but no growth in the dark in the inorganic autotrophic medium).

**Figure 5:**
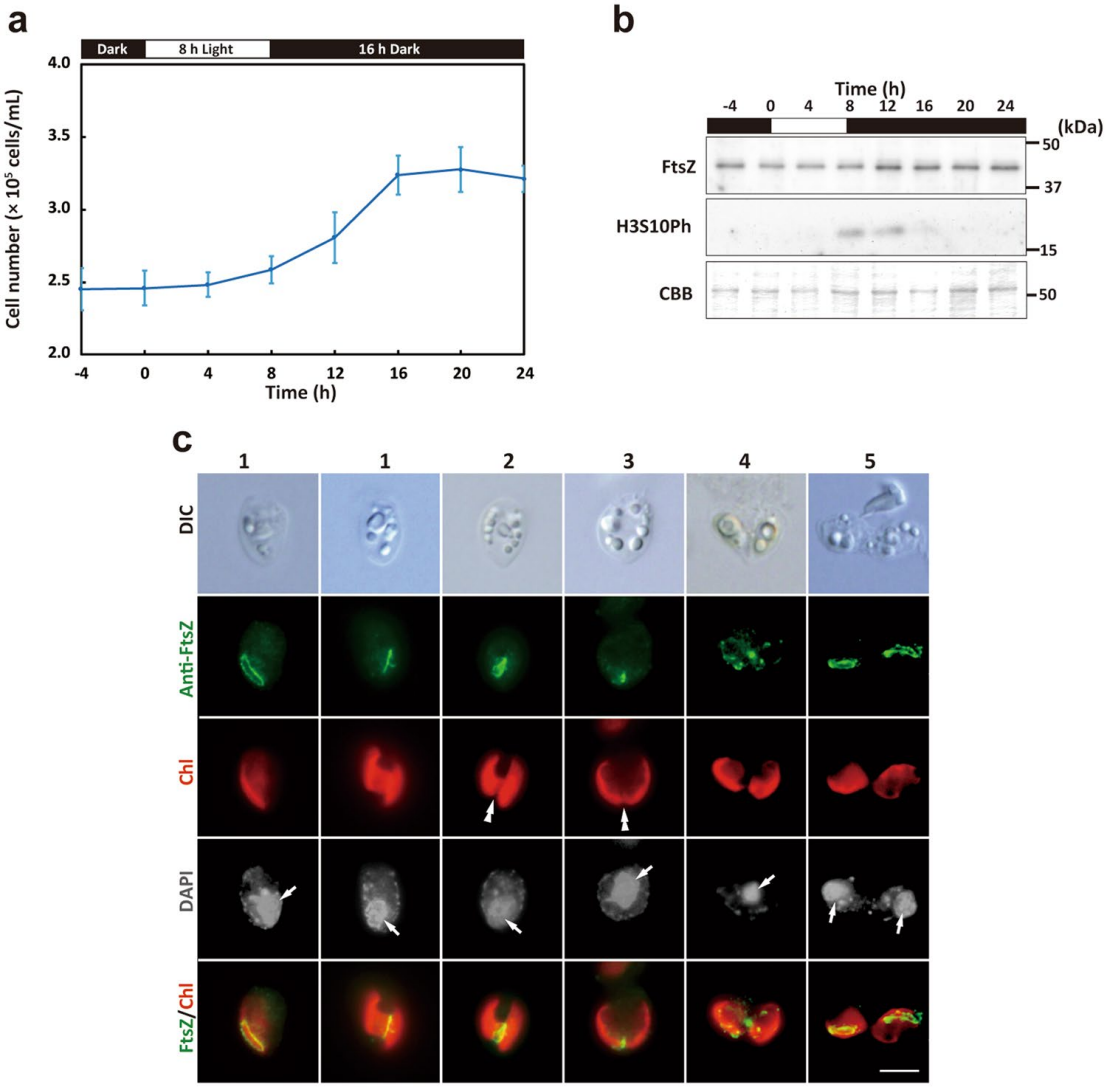
Change in the level and localization of nucleomorph-encoded FtsZ in the synchronous *G*. *theta* culture. (**a**) Change in the cell density in the culture synchronized by the 8-h light/16-h dark cycle. The error bar represents standard deviation of six technical replicates. (**b**) Immunoblot analysis showing the change in the FtsZ protein level and the level of histone H3 that is phosphorylated at serine 10 (H3S10ph) in the synchronous culture. Coomassie Brilliant Blue (CBB) staining is shown as the loading control. (**c**) Immunofluorescent micrographs showing the localization of FtsZ in the synchronized cells. DIC, images of differential interference contact; Anti-FtsZ, nucleomorph-encoded FtsZ detected with the antibody; Chl, chloroplast autofluorescence; DAPI, DNA stained with DAPI; FtsZ/Chl, merged images of Anti-FtsZ and Chl. The double arrowhead indicates constriction of the chloroplast division site. The arrow indicates DAPI signal of the nucleus. Scale bar = 5 µm.

The immunoblot analysis showed that the cellular FtsZ protein level was almost constant throughout the synchronous culture (Fig. 5a,b), while the M-phase protein marker H3S10ph exhibited time-dependent oscillation (detected at hour 8 and 12; Fig. 5a,b). Immunofluorescence microscopy showed that FtsZ localized in the chloroplast throughout the cell cycle progression (Fig. 5c). In all of the interphase cells observed, FtsZ localized at the broad bridge as a ring-like structure (Fig. 5c, stage 1). During chloroplast division, the FtsZ ring constricted (Fig. 5c, stages 2 and 3). In the cells, in which chloroplast division was completed, the FtsZ fluorescent signals were detected as dots dispersed throughout the chloroplast (Fig. 5c, stage 4). During cytokinesis, FtsZ again exhibited ring-like localization in both daughter chloroplasts (Fig. 5c, stage 5). These results suggest that expression of the nucleomorph-encoded FtsZ protein and its mRNA are not linked to the host cell cycle. This contrasts with nucleus-encoded FtsZ genes/proteins in primary algae, including the red algae, an ancestor of which was the origin of the nucleomorph and chloroplast in cryptophytes.

## Discussion

### Regulation of the nucleomorph replication cycle by the host cell cycle

The nucleomorph genome in cryptophytes encodes histone H2B, H3 and H4, but not H2A^19–22^. In this study, we have demonstrated that one of the four nucleus-encoded H2A localizes in the nucleomorph in *G*. *theta*. This situation is similar to the one in chlorarachniophyte *B*. *natans*, in which nucleus-encoded H2A and H2B are transported into the nucleomorph^16^. Thus, loss of H2A from the nuclear genome of the endosymbiont (the nucleomorph genome) and acquisition of nucleomorph-targeted H2A by the host nuclear genome occurred independently twice in the ancestors of cryptophytes and chlorarachniophytes.

The nucleomorph genome encodes certain cell-cycle-related proteins, such as CDC2, cyclin B and histone. In this study, we showed that the mRNA levels of *CYCLIN B* (also *CDC2* and alpha-tubulin) and histone genes were constant throughout the cell cycle (and nucleomorph replication cycle). In contrast, nuclear *CYCLIN B* mRNA exhibited G2/M-phase-specific accumulation as has been observed in other eukaryotes^24^. These results in the cryptophyte *G*. *theta* nucleomorph is similar to a recent report in the chlorarachniophyte *B*. *natans*, in which the mRNA levels of 99% of the nucleomorph genes remain constant throughout the cell cycle^17^. Thus, the loss of replication-cycle-linked transcriptional regulation in nucleomorphs is probably a common trait during the course of reductive evolution from nuclei to nucleomorphs.

In contrast to nucleomorph encoded histone genes, the nucleus-encoded nucleomorph *H2A* mRNA exhibited accumulation specifically around the nuclear S-phase, as in the case of the nuclear *H2A* in *G*. *theta* and nuclear histone genes in other eukaryotes^23^. These results suggest that transcriptional regulation in the host nucleus by the host cell cycle probably synchronizes the nucleomorph replication cycle to the host cell cycle.

The result of the specific expression of a nucleus-encoded nucleomorph protein in the cryptophyte *G*. *theta* is consistent with the recent reports in the chlorarachniophyte *B*. *natans* ^16, 17^. In *B*. *natans*, the nucleus-encoded nucleomorph *H2A* and *H2B* mRNA accumulate during the host S phase^16^. In addition, nucleus-encoded genes that encode proteins associated with the nucleomorph DNA replication (POLD, POLH, RFC1 and RPA1) are also transcribed during the host S phase^17^. Thus, nucleomorph replication came to be regulated at the host nuclear transcription level during the reduction of endosymbiotic nuclei to nucleomorphs in the course of evolution in both cryptophytes and chlorarachniophytes.

The nucleomorph genome does not encode any DNA polymerases in cryptophytes^19–22^, suggesting that a DNA polymerase(s) encoded by the host nucleus is targeted into the nucleomorph, as observed in chlorarachniophytes^17^. Identification and characterization of such nucleus-encoded nucleomorph DNA polymerase in cryptophytes will give further insights into the regulatory mechanism of the nucleomorph replication cycle by the host cell cycle.

### Regulation of the timing of chloroplast division by the host cell cycle

We previously showed that some, but not all, of the nucleus-encoded chloroplast division genes/proteins are specifically expressed in the S phase in primary algae^10^. In contrast, chloroplast-encoded division genes are expressed throughout the cell/chloroplast division cycle^10^. In stramenopiles, which possess secondary chloroplasts of a red algal origin but do not have nucleomorphs, FtsZ and DRP5B of a red algal endosymbiotic origin are encoded in the host nuclear genome^10, 27, 28^. *FtsZ* mRNA specifically accumulates during the S/G2 phase in the diatom (stramenopile)^15^.

In this study, the chloroplast-encoded *minD* and *minE* mRNA levels were constant throughout the cell cycle in the cryptophyte *G*. *theta*, as in the case of chloroplast-encoded division genes in primary algae. The nucleomorph-encoded *FTSZ* mRNA level slightly oscillated in the culture synchronized by a light/dark cycle, increasing in the light and decreasing in the dark (Fig. 3d). However, this oscillation was independent of cell cycle (and chloroplast division cycle) progression (Supplementary Information, Fig. S2b). Immunoblotting showed the FtsZ protein level to be almost constant throughout the cell cycle. FtsZ ring was detected in the chloroplast throughout the cell and chloroplast division cycle (Fig. 5c, stages 1, 2, 3, and 5) except that FtsZ dots spread throughout the chloroplast just after the completion of chloroplast division (Fig. 5c, stage 4). During cytokinesis, FtsZ again formed ring-like structure in the daughter chloroplasts (Fig. 5c, stage 5). Thus, the formation of the FtsZ ring is not sufficient to initiate the constriction of the chloroplast division site, and the timing of the initiation of the chloroplast division is the most likely regulated by nucleus-encoded proteins that are targeted to the chloroplast division site.

In this regard, any putative orthologs of the known chloroplast or cyanobacterial division proteins, including DRP5B, are not encoded in the nuclear genome of the cryptophyte *G*. *theta* ^11^. Thus, cryptophytes probably evolved as-yet-unidentified chloroplast division protein(s) that is encoded in the nuclear genome to regulate the timing of chloroplast division.

In contrast to cryptophytes, the nucleomorph genome of the chlorarachniophyte *B*. *natans* does not encode any putative chloroplast division proteins. Two FtsZ proteins (FtsZD-1 and FtsZD-2) are encoded in the nuclear genome and the respective mRNA level oscillates during cell cycle progression, although the peaks of the mRNA levels are not consistent with the timing of chloroplast division^11^. Immunofluorescence microscopy showed that both of the FtsZ proteins form a ring structure throughout the cell cycle^11^. Thus, as yet unidentified nucleus-encoded genes/proteins are transcribed/translated in a cell-cycle-dependent manner to initiate the chloroplast division in a specific period in the host cell cycle. As in the case of the cryptophyte *G*. *theta*, DRP5B and other putative orthologs of known chloroplast division proteins are not encoded in the nuclear genome of the chlorarachniophyte *B*. *natans* ^11^.

In this study, when the cell cycle progression was arrested in the early S phase by FdU treatment, chloroplast division was also blocked in *G theta* (Supplementary Information, Fig. S2c). This result is likely because the initiation of chloroplast division requires cell cycle progression. However, we can not rule out the possibility that the blockage of chloroplast division resulted form the blockage of chloroplast DNA replication by FdU.

### Comparison of the regulatory mechanisms of endosymbiotic cell division by the host cell cycle

In the case of both the primary and secondary endosymbiotic events, the cell division cycle of an endosymbiont came to be synchronized to the host cell cycle. However, there is a significant difference between the primary and the secondary acquisition of chloroplasts: i.e. between the cell division cycle of a bacterium (prokaryote) in the primary endosymbiotic event or that of a eukaryote in the secondary endosymbiotic event that came to be regulated by the eukaryotic host cell cycle.

**Figure 6:**
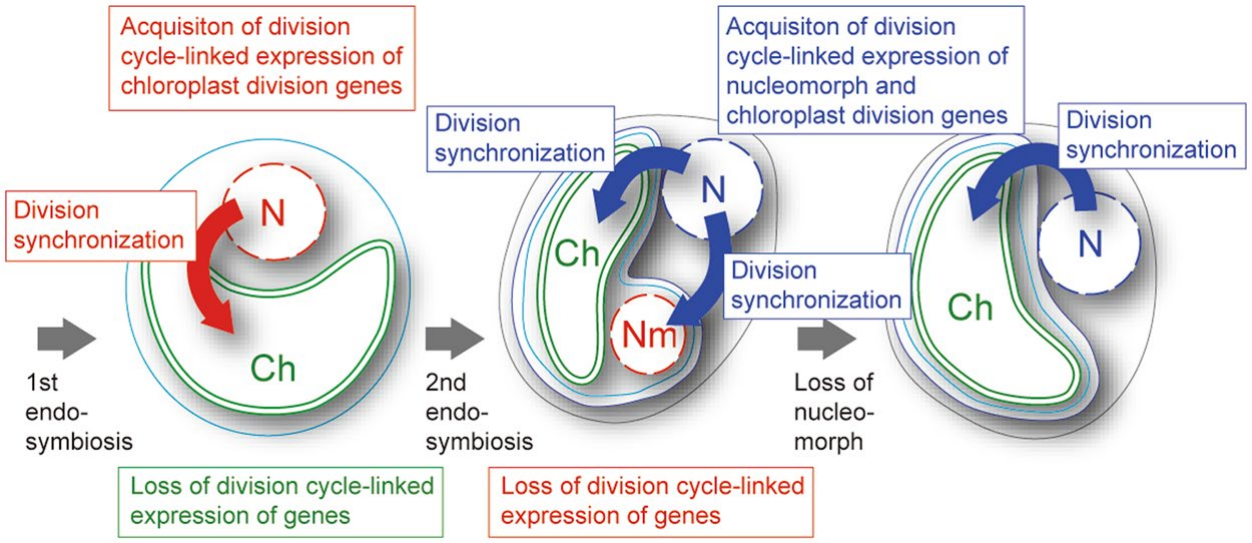
Schematic diagram of the evolution of the regulation of nucleomorph and chloroplast division by the host cell. “N”, “Nm” and “Chl” indicate nucleus, nucleomorph and chloroplast, respectively. Red and blue arrows indicate chloroplast division or nucleomorph-replication proteins that are encoded in the genome of the primary algal nucleus and the secondary algal nucleus, respectively.

Despite this difference, previous studies in primary algae, recent studies in the chlorarachniophyte *B*. *natans* ^11, 17^ and the present study in the cryptophyte *G*. *theta* all suggest that there is a common pathway in the evolution of the host-endosymbiont division synchronization. (1) Some of the chloroplast/cell division genes that had been transferred from the endosymbiont to the host nucleus or newly developed in the host nuclear genome came to be regulated by the host cell cycle progression (Fig. 6). This is the case for most of the nucleus-encoded primary chloroplast division genes. This is also the case for the nucleus-encoded *FtsZ* in diatoms. In addition, it is also predicted that transcription of as-yet-unidentified nucleus-encoded chloroplast division genes are regulated by the host cell cycle in chlorarachniophytes and cryptophytes, as discussed above. (2) The endosymbiotic genome has lost its division-cycle-linked transcriptional regulation of the chloroplast/cell division genes. This is the case for chloroplast-encoded division genes in both chloroplasts of primary and secondary endosymbiotic origin. This is also the case for nucleomorph-encoded cell-cycle-related genes.

Further studies, especially identification and characterization of the as yet unidentified nucleus-encoded chloroplast division proteins in cryptophytes and chlorarachniophytes, will give significant insights into the evolution of the division synchronization mechanisms in secondary chloroplasts.

## Methods

### Targeting signal prediction and phylogenetic analyses

The amino acid sequences of *G*. *theta* histone H2A were obtained from the JGI portal website (http://genome.jgi.doe.gov/Guith1/Guith1.home.html). The signal peptide cleavage site was predicted by SignalP 3.0^29^. The 41 H2A amino acid sequences were aligned and the poorly aligned regions were eliminated according to Hirakawa *et al*.^16^, except for the following, a setting of Gblocks^30^ was applied; half of the gapped positions allowed, the minimum number of a conserved and a flank positions set to 30% and 28% of the number of taxa plus one, respectively. The aligned sequences were calculated using RAxML v8.0.0^31^ with an LG + GAMMA model selected by Aminosan^32^, and the corresponding bootstrap support values were calculated through ML analysis of 1,000 pseudoreplicates. Bayesian analysis was performed with MrBayes5D^33^ using an LG + GAMMA model. Markov chain Monte Carlo iterations were carried out until 20,000,000 generations were attained. Trees were sampled every 100 generations. The first 200,000 generations were discarded as burn-in. Posterior probabilities were calculated from all of the post burn-in trees.

### Algal culture

*G*. *theta* CCMP2712 was obtained from The National Center for Marine Algae and Microbiota. The cells were cultured in h/2 medium (an inorganic photoautotrophic medium) at 20 °C under illumination (50 µE m^−2^s^−1^) in Erlenmeyer flasks without agitation. To synchronize *G*. *theta*, the cells in log phase were diluted with fresh medium and transferred to dark for 24 h to arrest the cells in the G1 phase. 500 mL of culture in an Erlenmeyer flask were subjected to an 8-h light/16-h dark cycle (50 µE m^−2^s^−1^) at 20 °C. To arrest the cells in S-phase, a 1/1,000 volume of 100 mg/mL 5-fluorodeoxyuridine (FdU) dissolved in water was added at the onset of the light period.

### Preparation of antibodies

The antibodies against *G*. *theta* nucleomorph histone H2A and FtsZ were raised in rabbits using the respective recombinant proteins. The cDNA encoding the full length (nucleomorph histone H2A) or a partial fragment (FtsZ) of the respective protein was amplified by PCR using the primers listed in Supplementary Table 2. In the case of *FTSZ*, a region well conserved in cryptophytes was amplified from cDNA of *Chroomonas mesostigmatica* CCMP1168. Subsequent procedures (cloning, polypeptide expression and antibody purification) followed the methods in Miyagishima *et al*.^10^.

### Immunofluorescence microscopy

*G*. *theta* cells were collected by centrifugation and fixed with fixation buffer (3% paraformaldehyde, 50 mM Pipes, pH 6.8, 10 mM ethylene glycol tetraacetic acid, 5 mM MgSO_4_ and 0.3 M sucrose) for 30 min at room temperature. The fixed cells were permeabilised with 0.5% Triton X-100 in PBS for 15 min, and then washed three times with PBS supplemented with 0.01% Tween 20 (PBS-T). After blocking with the blocking buffer (2% bovine serum albumin in PBS-T) for 30 min, cells were labeled with the anti-FtsZ antibody or anti-nucleomorph H2A antibody diluted to 1:1,000 in the blocking buffer for 2 h at room temperature. Primary antibodies were detected with Alexa Fluoro 488 goat anti-rabbit IgG (Thermo Fisher Scientific) at a dilution of 1:2,000 in the blocking buffer for 1 h at room temperature. The cells were observed under an Olympus BX51 and the images were captured with an Olympus DP71.

### Immunoblot analyses

Cells were harvested from 2 mL of the synchronous culture at the indicated time points by centrifugation. Cells were then resuspended in the SDS-PAGE sample buffer (50 mM Tris, pH 6.8, 6% 2-mercaptoethanol, 2% sodium dodecyl sulfate, 10% glycerol). Protein content was quantified with XL-Bradford (APROSCIENCE), and equal amounts of the total protein were subjected to immunoblot analysis. Immunoblotting was performed as described in Miyagishima *et al*.^34^ using 12% SDS-polyacrylamide gels. The anti-FtsZ, anti-nucleomorph H2A or anti-H3S10Ph^35, 36^ antibodies was diluted to 1:1000. The primary antibodies were detected by HRP-conjugated goat anti-rabbit antibody (Thermo Scientific) diluted to 1:20,000.

### Quantitative reverse transcription- (RT-) PCR

Cells were harvested from 20 ml synchronized culture by centrifugation and stored at −80 °C until RNA extraction. Total RNA was extracted with NucleoSpin® RNA XS (TaKaRa) according to the manufacturer’s instructions. cDNA was synthesised from 300 ng of total RNA using a mixture of a random hexamer and oligo dT primer (1:9 in ratio of concentration) with PrimeScript RTase (TaKaRa).

The quantitative PCR analyses were performed using a StepOne-Plus Real-Time PCR system (Life Technologies) and Power SYBR Green Master Mix (Life Technologies). The values of the respective genes were normalised with the data on 18S ribosomal RNA. The primer sequences are shown in Supplementary Table 2.

### EdU labelling assay

Cells were synchronized in a multi-well plate by an 8-h light/16-h dark cycle (50 µE m^−2^s^−1^) at 20 °C. 4 mL of synchronized culture was incubated with a 1/100 volume of 10 mM EdU dissolved in DMSO for 2 h at the indicated time points. After the incubation, cells were harvested by centrifugation and fixed with the fixation buffer (same as in the immunofluorescence microscopy) for 30 min at room temperature. The fixed cells were permeabilized with 1% Triton X-100 in PBS for 30 min. The cells were washed two times with 2% bovine serum albumin in PBS-T and then were incubated with the reaction cocktail of Click-iT EdU Alexa Fluor 488 Imaging Kit (Thermo Fisher Scientific) for 30 min.

## Electronic supplementary material

Supplementary Infomation
