# Supplementary material for: Regulation of chloroplast and nucleomorph replication by the cell cycle in the cryptophyte Guillardia theta
Source: Sci Rep. 2017 May 24;7:2345. doi: 10.1038/s41598-017-02668-2 (PMC5443833; doi:10.1038/s41598-017-02668-2)
Supplement: Supplementary file 1 — Supplementary Infomation [file 41598_2017_2668_MOESM1_ESM.pdf]

SUPPLEMENTARY INFOMATION

Regulation of chloroplast and nucleomorph replication by the cell cycle in the cryptophyte *Guillardia theta*

Ryo Onuma[1] *, Neha Mishra[1,2], Shin-ya Miyagishima[1,2] *

[1]Department of Cell Genetics, National Institute of Genetics, Yata 1111, Mishima, Shizuoka 411-8540, Japan

[2]Department of Genetics, Graduate University for Advanced Studies (SOKENDAI), Mishima, Shizuoka 411-8540, Japan

*Correspondence: Ryo Onuma and Shin-ya Miyagishima, Department of Cell Genetics, National Institute of Genetics, Yata 1111, Mishima, Shizuoka, 411-8540, Japan.

E-mail; ronuma@nig.ac.jp and smiyagis@nig.ac.jp

Tables

Supplementary Table 1. GI number of chloroplast division proteins in cyanobacteria, primary algae, land plant and secondary algae.

**Organisms**	**Strains**	**Protein**									
		FtsZ	ARC6	FtsW	SepF	MinC	MinD	MinE	DPR5B	PDV	PDR1
Cyanobacteria	*Synechocystis* sp. PCC 6803	1652575	1001436			14595186	1001783	1001782			
Cyanophora paradoxa	*Cyanophora paradoxa* UTEX555	66954464		11467412	11467353		383212634	383212636			
Cyanidioschyzon merolae	*Cyanidioschyzon merolae* 10D	34850216, 34850218							27544794		449019153
Chlamydomonas reinhardtii	*Chlamydomonas reinhardtii* CC-503 cw92 mt+	159488863, 159484937	159465413			159469133	159477869	159478206	159489354		
Chlorella vulgaris	*Chlorella vulgaris* C-27	384080863					524873	384080867	384080865		
Mesostigma viride	*Mesostigma viride* NIES-296	384080857		11466430			11466429		384080859		
Arabidopsis thaliana	*Arabidopsis thaliana* ecotype: Columbia	75220266, 75264335, 21903428	75333910				75264960	75168864	327507753	75171154, 75215718	
Guillardia theta	*Guillardia theta* CCMP2712	162606304					11467635	11467634			
Bigelowiella natans	*Bigelowiella natans* CCMP2755	JGI#39262, JGI#92991									
Thalassiosira pseudonana	*Thalassiosira pseudonana* CCMP1335	224005372, 224012130.					224003325		224002629		
Emiliania huxleyi	*Emiliania huxleyi* CCMP1516 and CCMP373	485623283, 485607205					71842293	485603575	485617808, 485627595		

Supplementary Table 2. Primers used in this study

Target Transcript	Genome	Sequence ID	Purpose	Primer sequences (5' to 3')
FTSZ	Nucleomorph	GI#13812269:16993..18192	cloning	CACC TCCCCTTGTTTGATTAAAGTAATTGG<br>TCAAAAACCAGTAGCTACAACAGTTATAG
Nucleomorph *H2A*	Nucleus	JGI#100176	cloning	GATCATCCCTTCACCATCTTTCTGGGGACC<br>GTTGAGCTCGCCCTTTGCTTCCGCCTTCTC
Nuclear *H2A*	Nucleus	JGI#47177	RT-PCR	TCTCGCTGCCGTTCTTGAG<br>AGCGTTGCCAGCCAACTC
Nuclear *CYCLIN A*	Nucleus	JGI#85274	RT-PCR	AGCAAGTCCAGCACGATATCAA<br>TCCACAAGCCAGTCGATGAG
Nuclear *CYCLIN B*	Nucleus	JGI#157506	RT-PCR	CGCCACCGGTGAACTACAT<br>CGCGCATCTTCTCGTTGA
Nucleomorph *HISTONE H2A*	Nucleus	JGI#100176	RT-PCR	GGAGGCGTCCTTCCAAACAT<br>CCCTTCCCGCTATCTTTCG
FTSZ	Nucleomorph	GI#13812269:16993..18192	RT-PCR	TGCAGCAGTTGCAGCTATAAGTT<br>CAATTCCTCTGGCTTTTTCGA
Nucleomorph *HISTONE H2B*	Nucleomorph	GI#13812269:27256..27564	RT-PCR	CACGGCTAGAGAAATACAAACTTCTG<br>TCGGATACTGAATGCTTTGCAA
Nucleomorph *CYCLIN B*	Nucleomorph	GI#13812105:74047..75090, complement	RT-PCR	GCTTCCAAATACGAAGAAATATATGCT<br>TGACTGTGGTGATGGTAAATTAAATT
Nucleomorph *CDC2*	Nucleomorph	GI#13812269:131401..132288, complement	RT-PCR	CATTTGGTTGTGTTATAGGAGAATTGA<br>TGTGGTAGGAGTTCCAATAGTCTGAA
Nucleomorph *TUBA*	Nucleomorph	GI#13812269:29716..31062	RT-PCR	TGTGGGCGAAGGAATGGA<br>TCTCAAGTGCCGCCATATCTT
MIND	Chloroplast	GI#11467607:25272..26081, complement	RT-PCR	ATCTGGTGAATGTCGGCTAGAAC<br>TGCTGCAGGTAATAAAACTAAATTTGG
MINE	Chloroplast	GI#11467607:24982..25248, complement	RT-PCR	TCTGAAGTTAGTGTTGGCACATGA<br>TCTTCACGCATTTTTTCTAAGGTAGA
18S rRNA	Nucleus	JGI#Guith1_scaffold_88:383-2155,complement	RT-PCR	CGACTCACGGAGGGTTGTATTT<br>GCGGTTCGTTTAGTTACTATGAATCA

Figures

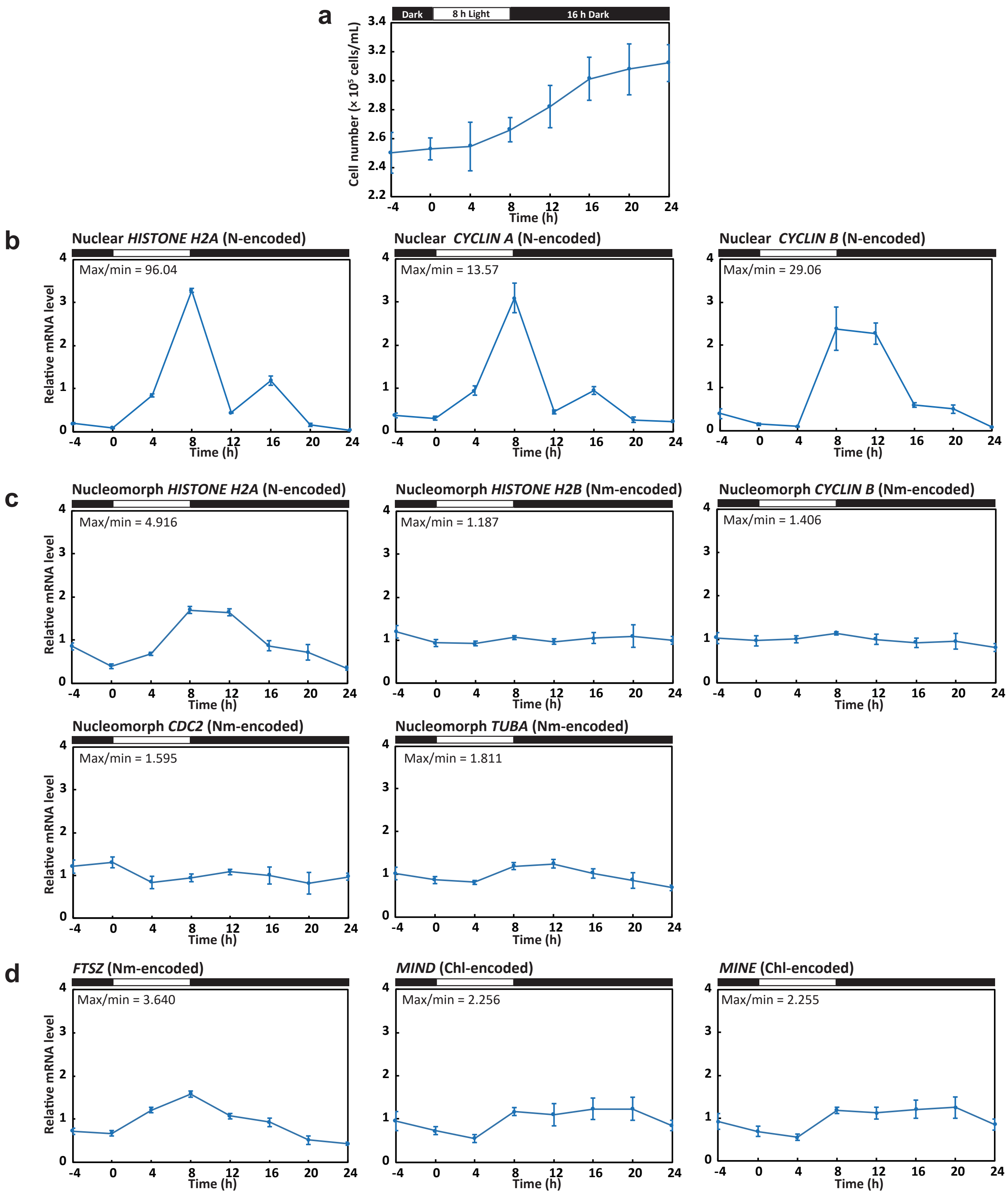

Supplementary Figure 1. A biological replicate of the quantitative RT-PCR shown in Figure 3.

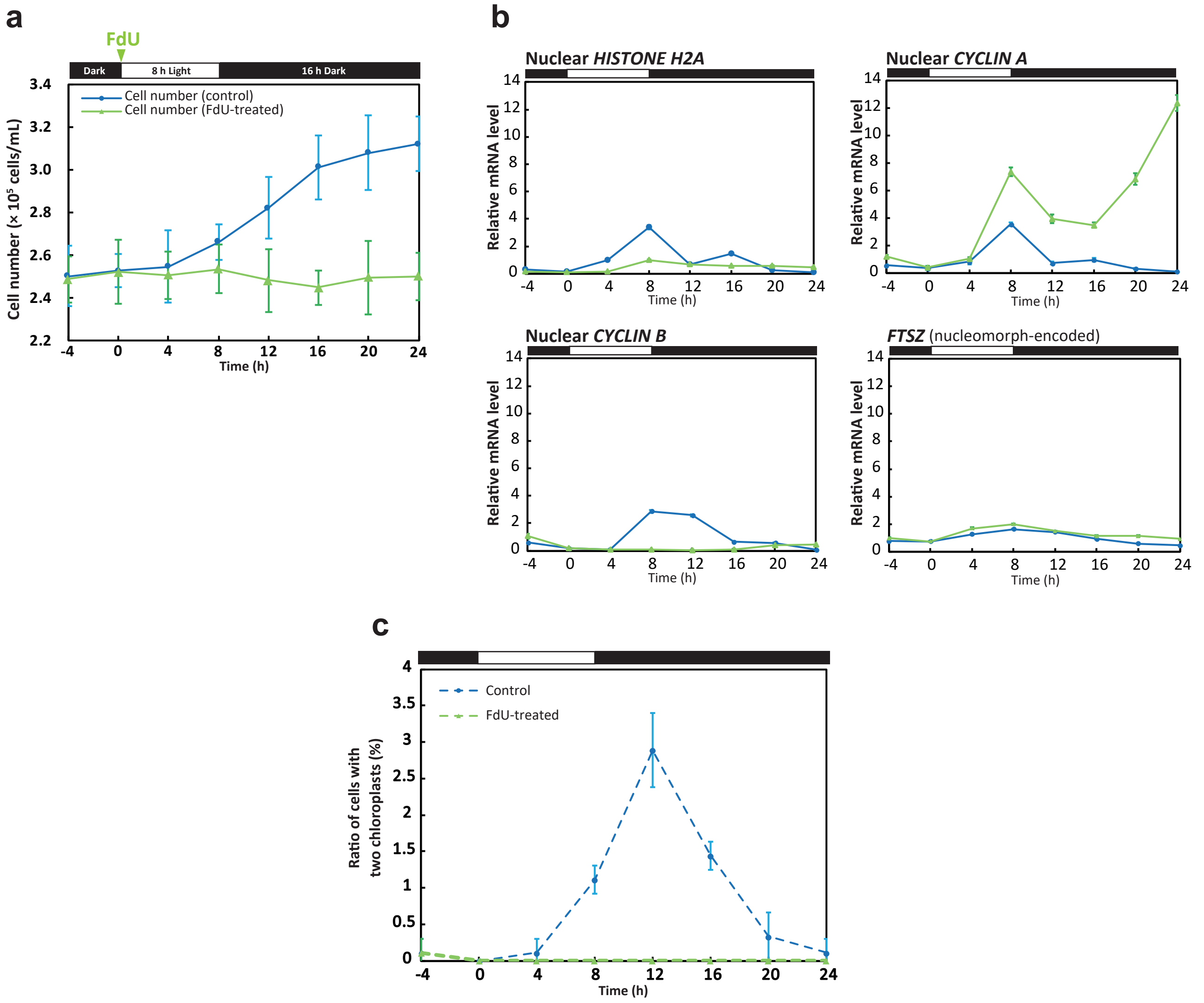

Supplementary Figure 2. The effect of S-phase arrest with FdU on the change in mRNA levels of cell cycle/chloroplast division gene in the synchronous *G. theta* culture. (a) Change in the cell density in the culture synchronised by an 8-h light/16-h dark cycle without (blue) or with FdU treatment (green). FdU was added at the onset of the light period. The error bar represents standard deviation of six technical replicates. **(b)** Quantitative RT-PCR analyses showing the change in the mRNA levels of S-phase markers nuclear *HISTONE H2A* and *CYCLIN A*, M-phase marker *CYCLIN B* and nucleomorph-encoded *FTSZ*. The values in the control (blue) and FdU-treated (green) synchronous cultures are shown. 18S ribosomal RNA was used as the internal control. The average value from hour 0 to 20 in the control culture was defined as 1.0 for each mRNA. The error bar represents standard deviation of three technical replicates. **(c)** Change in the frequency of cells with two divided chloroplasts in the control (blue dashed line) and FdU-treated (green dashed line) synchronous cultures. The error bar represents standard deviation of three biological replicates.
